# Antimicrobial susceptibility patterns of commensal fecal bacteria isolated from pigs with an intentional genomic alteration that included the selectable marker gene *nptII*

**DOI:** 10.3389/fmicb.2026.1885937

**Published:** 2026-08-25

**Authors:** Tessa E. LeCuyer, Jeffrey Monahan, Kayla Farrell, Kent Adams, Anneke Walters, Diamond McClendon, Alexandra Fox, John R. Bianchi

**Affiliations:** 1 Department of Pathology, Microbiology, and Immunology, UC Davis Weill School of Veterinary Medicine, UC Davis, Davis, CA, United States; 2 United Therapeutics Corporation, Department of Xeno R&D, Blacksburg, VA, United States; 3 Virginia Tech Animal Laboratory Services, Virginia-Maryland College of Veterinary Medicine, Virginia Polytechnic Institute and State University, Blacksburg, VA, United States

**Keywords:** alpha-gal, antimicrobial resistance (AMR), antibiotic resistance, genetically engineered animals, NARMS

## Abstract

**Introduction:**

Animals with intentional genomic alterations (IGAs) hold promise for meeting increasing worldwide demand for animal-source proteins. As part of regulatory risk assessment for introducing animals with IGAs into the food chain, monitoring commensal bacterial microbiota is recommended due to concern that antimicrobial resistance genes used during IGA selection could be transferred, via horizontal gene transfer, to gastrointestinal or environmental bacterial populations, potentially contributing to antimicrobial resistance. The objective of this study was to assess the antimicrobial susceptibility patterns in commensal bacteria isolated from fecal samples of GalSafe™ pigs that have an IGA that includes the aminoglycoside resistance gene *nptII*.

**Methods:**

Antimicrobial resistance rates observed in *Escherichia coli*, *Salmonella*, *Campylobacter* and *Enterococcus* isolated from GalSafe™ pigs were compared to resistance rates observed in conventional pigs at slaughter. Bacterial isolates were tested for antimicrobial resistance genes by PCR and one isolate underwent whole genome sequencing.

**Results:**

In total, 137 bacterial isolates recovered from 55 fecal samples collected from 47 individual adult GalSafe™ pigs were evaluated. Prevalence of antimicrobial resistance in GalSafe™ pigs was generally similar to, or lower than, resistance prevalence reported from conventional pigs at slaughter, based on National Antimicrobial Resistance Monitoring System (NARMS) data. Higher resistance rates in GalSafe™ pigs were observed only for quinolones in *Campylobacter coli* (ciprofloxacin and nalidixic acid) and nitrofurantoin in *Enterococcus* spp. One isolate (*E. coli*) was positive for *nptII* neomycin resistance gene, the same gene used for IGA selection in GalSafe™ pigs, and the remaining 136 isolates were negative for *nptII*. However, the positive isolate did not appear to contain nptII derived from the GalSafe™ pig genome as the sequences flanking the gene did not match the IGA.

**Discussion:**

We did not detect evidence of *nptII* gene transformation into bacterial species of potential human health importance in this population of GalSafe™ pigs.

## Introduction

Animals with intentional genomic alterations (IGAs) allow for precise selection for traits that increase production value. The GalSafe™ pig originated from genetic engineering to eliminate expression of the α-galactosidase epitope, a major immunogenic carbohydrate responsible for hyperacute immune rejection in xenotransplantation and the trigger for α-gal syndrome (Cabezas-Cruz et al., 2022; Chung et al., 2008; Commins et al., 2011; Phelps et al., 2003; Platts-Mills et al., 2020). Production of pigs deficient in α1,3galactosyltransferase (GGTA1), the enzyme responsible for α-gal synthesis, eliminates α-gal expression in swine (Phelps et al., 2003). This *GGTA1* knockout approach is the foundation for α-gal–deficient pig lines, including the GalSafe™ pig.

Establishment of the *GGTA1* knockout genetic modification employed a selectable marker gene, neomycin phosphotransferase II gene (*nptII*), which encodes the APH(3’)-IIa enzyme that confers resistance to aminoglycoside antibiotics such as kanamycin and neomycin (Food and Drug Administration (FDA), 2020a). In GalSafe™ pigs, a promoterless internal ribosome entry site–neo–polyA cassette was inserted as a gene trap into the *GGTA1* locus via homologous recombination, disrupting *GGTA1* transcription while permitting *nptII* expression under control of the endogenous *GGTA1* promoter. Therefore, GalSafe™ pigs have inactivated α-gal synthesis and a single integrated *nptII* marker gene. The GalSafe™ pig is approved by the U.S. Food and Drug Administration for use as human food and as a source of materials for human therapeutics, including medical devices, drugs, biologics, and excipients (Food and Drug Administration (FDA), 2020a).

The use of antibiotic resistance genes in genetically engineered animals has raised biosafety and regulatory concerns, primarily related to the theoretical potential for horizontal gene transfer of resistance determinants, such as *nptII*, to commensal or environmental bacteria, which could contribute to antimicrobial resistance (AMR; Kaeppler, 2000; Kleter and Kuiper, 2002). Although this risk is considered low, regulatory agencies have emphasized the importance of post approval monitoring to ensure that antibiotic resistance marker genes do not disseminate in unintended ways following commercialization. The objective of this study was to assess AMR patterns in commensal bacteria isolated from fecal samples of GalSafe™ pigs, with a secondary aim of contextualizing these findings by comparison to antimicrobial resistance rates observed in conventional pigs at slaughter, as reported by the National Antimicrobial Resistance Monitoring System (NARMS).

## Materials and methods

### NARMS data

Data on antimicrobial resistance from conventional pigs was downloaded from NARMS in the form of an Excel spreadsheet (Food and Drug Administration (FDA), 2025a). Aggregate NARMS data was evaluated for *Enterococcus* spp., *E. coli*, *Salmonella*, and *Campylobacter* spp. NARMS data from the period of 2013–2019 was available and included in the study. Global Resistome Data was accessed through the NARMS website platform (Food and Drug Administration (FDA), 2025b) using whole genome sequencing data in NCBI using queries for *E. coli* and *Salmonella* isolated from pigs and pork products through the NARMS surveillance system.

### Fecal specimens

A total of 55 fecal samples were collected from 47 individual GalSafe™ pigs at least 117 days of age, 23 males and 24 females, between April and September 2022. The sampled animals were all from at least the tenth generation of GalSafe™ pigs. GalSafe™ pigs have had a closed breeding population for approximately 17 years, and aminoglycoside antimicrobials are not used to comply with regulatory requests. No animals undergoing active medical treatment were included in the study. Fecal samples were collected directly from the rectum of each pig with a gloved hand, and fecal samples were shipped overnight on ice packs to Virginia Tech Animal Laboratory Services (Blacksburg, VA). All samples were processed for fecal culture within 24-h of collection using routine laboratory methods. Because the total number of available animals was small, seven animals had multiple fecal samples tested; one animal had three specimens collected, and six animals had two specimens collected during the study period (see Supplementary Table 3 for more information on isolates from repeated animals).

### Bacterial isolation

Fecal samples were plated directly onto Columbia blood agar (Remel, Thermo Fisher Scientific, Lenexa KS), bile esculin agar (Hardy Diagnostics, Santa Maria, CA), MacConkey agar (Remel, Thermo Fisher Scientific, Lenexa KS), CVA (Hardy Diagnostics, Santa Maria, CA), XLT4 (Hardy Diagnostics, Santa Maria, CA), and 1 g of feces was placed into tetrathionate broth activated with iodine (Hardy Diagnostics, Santa Maria, CA). *Salmonella* enrichment occurred for 24 h at 37 °C in tetrathionate broth, and then the broth was subcultured to XLT-4 agar, MacConkey agar, and RV broth (Hardy Diagnostics, Santa Maria, CA). RV broth was subcultured after an additional 18–24 h incubation onto XLT-4 and MacConkey agars. Isolates with morphologic characteristics consistent with *Enterococcus* spp., *Salmonella* spp., *Escherichia coli*, and *Campylobacter* spp. were identified by matrix-assisted laser desorption/ionization time-of-flight mass spectrometry (MALDI-TOF, Bruker Biotyper, Bruker Daltonics, Billerica MA). The most predominant isolate of each genus of interest was banked at −80 °C in tryptic soy broth or brain heart infusion broth with 15% glycerol (Hardy Diagnostics, Santa Maria, CA) and used for additional testing.

### Susceptibility testing

Antimicrobial susceptibility testing was performed by broth microdilution to determine minimum inhibitory concentrations (MIC) of drugs using commercially available plates (Trek Diagnostics, Thermo Fisher Scientific, Oakwood Village, OH) and for kanamycin by using gradient MIC strips (Liofilchem, Waltham MA). The Gram Negative NARMS panel (CMV5AGNF) was used for *Salmonella* and *E. coli*. The Gram Positive NARMS panel (CMV4AGPF) was used for *Enterococcus* spp. and the *Campylobacter* NARMS panel (CAMPY2) was used for *Campylobacter* spp. NARMS breakpoints were used to categorize isolates as susceptible, intermediate or resistant based on MIC values (Centers for Disease Control and Prevention (CDC), 2025; Food and Drug Administration (FDA), 2017). Differences between the methods used for susceptibility testing compared to the NARMS method of antimicrobial susceptibility testing were: higher transfer volume (30 ul) of bacterial suspension for *Enterococcus* spp. for increased sensitivity for detection of hetero-resistance, no documentation of time to incubation of susceptibility test plates, and enumeration of bacterial colony forming unit counts from inoculum suspensions on a routine, but not weekly, basis. Other methods were aligned with NARMS antimicrobial susceptibility test methods (Food and Drug Administration (FDA), 2020b). Quality control testing of all antimicrobials used in this study was performed prior to performing broth microdilution and gradient strip testing on the fecal isolates and testing only proceeded if MICs of quality control organisms were within range.

### Resistance gene PCR

Frozen bacterial isolates suspended in either tryptic soy broth with 15% glycerol, brain heart infusion broth with 15% glycerol (*Campylobacter* spp.) or Mueller-Hinton broth were transferred to Revivicor (Blacksburg, VA) for DNA extraction and PCR testing for AMR genes. Total Bacterial (genomic and plasmid) DNA was extracted using a commercial kit (DNeasy/QIAamp, Qiagen, Germantown, MD) according to manufacturer’s instructions, including the manufacturer’s recommended modified method for the isolation of DNA from Gram-positive bacteria (*Enterococcus*), which requires user-supplied enzymatic lysis buffer prepared as described by the manufacturer. A multiplex PCR assay (EmeraldAmp GT Master Mix, TaKaRa Bio, San Jose CA) was used to identify *aaaC1*, *nptII*, *bla*, and *aph* genes, along with a 16S rRNA bacterial genomic positive control. Primers for the multiplex PCR are described in Supplementary Table 1, including neomycin primers that were designed for this study (Nadkarni et al., 2002; Rinttilä et al., 2004). A secondary assay was designed to detect a 631 bp fragment of the internal ribosome entry site-neo sequence as it exists in GalSafe™ genomic DNA (primer pair 657F8580 and 657R9116) along with primers for the bacterial 16S rRNA positive control (Supplemental Table 1). Thermocycling conditions for both assays were an initial denaturing for 60 s at 98 °C, followed by 30 cycles of 10 s at 98 °C for denaturation, 30 s at 63 °C for annealing, 60 s at 72 °C for extension and a final extension cycle of 60 s at 72 °C. As positive control for the resistance genes, a 3768 bp plasmid, pGANK-PC, containing primer and template sequence of four antibiotic resistance genes (gentamicin, ampicillin, kanamycin, and neomycin) was synthesized [Integrated DNA Technologies; San Diego, CA (IDT)]. Three gene fragments, gentamicin resistance (GmR) sequence from *aacC1* gene, ampicillin (AmpR) resistance sequence from *bla* gene, neomycin resistance sequence from *nptII* gene were synthesized and integrated into IDT cloning vector, pUCIDT-KAN that already contained the kanamycin resistance (KanR) gene sequence.

The multiplex PCR reaction could detect 100 copies of *nptII, bla, aph*, and *aaaC1* in the presence or absence of fecal bacterial DNA, indicating that the assay was adequately sensitive to detect the quantities of each gene isolated from a pure culture bacterial isolate.

### Whole genome sequencing

One *E. coli* isolate that was positive by PCR for *nptII* underwent long-read nanopore whole genome sequencing. A library was prepared from the same DNA specimen used for resistance gene PCR using the Ligation sequencing DNA V14 kit (SQK-LSK114, Oxford Nanopore Technologies, Oxford UK) following manufacturer’s instructions. The library was run on a FLO-MIN114 flow cell using a MinION device (Oxford Nanopore Technologies). Base calling was conducted during the sequencing run using the MinKNOW software (Oxford Nanopore Technologies; v25.05.14) with the high-accuracy model, 400 bps. A min Q score of 9 was required for base calling and BAM files were generated every hour of the run, which were subsequently used for alignment.

Epi2ME software (Oxford Nanopore Technologies) was used to align Nanopore-generated BAM files to the pPL657 reference sequence (wf-alignment; version 1.2.3) and the GenBank U00004.1 reference sequence (wf-alignment; version 1.2.6). Default parameters for the wf-alignment were used, Integrative Genomics Viewer (IGV) was selected, depth coverage was selected, Minimap2 (parameter preset = dna, Threads = 4) and the BAM files generated from the alignment were visualized in IGV to conduct secondary confirmation. The sequence is available in GenBank (BioProject PRJNA1466911).

### Statistics

*A priori* sample size was determined using an exact binomial test, comparing the percent resistance to gentamicin between groups, with alpha = 0.05, power = 0.8, and constant proportion = 0.03, based on composite gentamicin resistance prevalence in NARMS data for *Salmonella* spp., *E. coli*, *Enterococcus* spp. and *Campylobacter* spp., using G*Power software (Kang, 2021). To detect a resistance rate difference of at least 10% between GalSafe™ and conventional pigs, a sample size of at least 21 isolates from each group was required. Resistance rates for each antimicrobial were compared by two-tailed exact binomial tests in R Studio 2022.07.0 using baseline R version 4.2.3 functions for exact binomial tests (using NARMS data as a reference distribution for expected resistance prevalence) with Holm-Bonferroni adjustment for multiple comparisons of resistance rates across drugs within each genus (R Core Team, 2024). Prevalence of *nptII* in GalSafe™ pig *E. coli* compared to NARMS Resistome data was compared by Fisher’s exact test in R version 4.2.3 (R Core Team, 2024; Food and Drug Administration (FDA), 2025b).

## Results

A total of 137 bacterial isolates from the target genera were isolated from 55 fecal samples. The numbers of organisms isolated from each genera of interest are in Table 1. The prevalence of antimicrobial resistance that was significantly different from the prevalence of resistance in conventional pigs included in the NARMS dataset is in Table 2. Full antimicrobial resistance information for all organisms and all drugs tested is available in Supplementary Table 2.

**TABLE 1:** Identification of species and genus for the 137 *Enterococcus*, *Escherichia*, *Salmonella* or *Campylobacter* isolated for further analysis from 55 fecal samples.

Genus	Species	No. colonies isolated
*Enterococcus*	*faecium*	12
	*hirae*	36
	Total	48
*Escherichia*	*coli*	55
	Total	55
*Salmonella*	Total	3
*Campylobacter*	*coli*	28
	*jejuni*	0
	*lanienae*	3
	Total	31

**TABLE 2:** Prevalence of antimicrobial resistance in fecal bacteria isolated from GalSafe™ pigs and cecal bacteria isolated from conventional pigs by the NARMS program.

Organism	Drug	GalSafe (No. R/total N)	NARMS (No. R/total N)	GalSafe resistance proportion (95% CI)	Reference NARMS resistance proportion	*p*-value (binomial)	Holm adjusted *p*-value (binomial)
*Campylobacter coli*	Ciprofloxacin	10/28	281/2131	0.357 (0.186–0.559)	0.132	0.002	0.017
*Campylobacter coli*	Nalidixic acid	10/28	283/2131	0.357 (0.186–0.559)	0.133	0.002	0.017
*Enterococcus*	Linezolid	1/48	0/3060	0.021 (0.001–0.111)	0	<0.001	<0.001
*Enterococcus*	Tetracycline	7/48	2244/3060	0.146 (0.061–0.278)	0.733	<0.001	<0.001
*Enterococcus*	Nitrofurantoin	7/48	17/3060	0.146 (0.061–0.278)	0.006	<0.001	<0.001
*Enterococcus*	Erythromycin	2/48	1101/3060	0.042 (0.005–0.143)	0.360	<0.001	<0.001
*Enterococcus*	Kanamycin	0/48	359/2034	0.000 (0.000–0.074)	0.176	<0.001	0.002
*Enterococcus*	Streptomycin	2/48	601/3060	0.042 (0.005–0.143)	0.196	0.003	0.034

No. R/Total *N*-values represent the number of resistant isolates over the total number of isolates tested. Exact binomial tests were used to compare GalSafe resistance proportions to NARMS reference rates with 95% confidence intervals (CI). *P*-values were adjusted within organism using the Holm method. This table includes organism and drug combinations that were significantly different in the GalSafe pigs sampled compared to the reference NARMS population (adjusted *p* < 0.05). Additional data is found in Supplementary Table 2.

### Repeat testing of select animals

Seven animals underwent repeated fecal sampling to assess the stability of the fecal microbiota of interest in GalSafe™ pigs over time. One animal had three specimens collected during the study period and six animals had two samples collected during the study period. Each collection occurred at least 6 weeks apart. All animals in this group had *E. coli* isolated from each fecal sample, but the presence of the other organisms of interest often varied over time. Only one of the seven animals that underwent repeat testing had the same composition of organisms recovered from all fecal samples (Supplementary Table 3).

### Antimicrobial resistance genes

All 137 unique bacterial isolates were tested for a panel of antimicrobial resistance genes by PCR. One *E. coli* isolate was positive for *nptII* neomycin resistance gene, and the remaining 136 isolates were negative for *nptII*. The isolate that was positive for *nptII* did not have amplification of the internal ribosome entry site on pPL657 that flanked *nptII* in the sequence that was used to develop the GalSafe™ pig, suggesting that the *nptII* sequence is not of GalSafe™ origin. Nine isolates had the *bla* ampicillin resistance gene; all were *E. coli* and all had phenotypic resistance to ampicillin. No isolates were positive for *aph*, the kanamycin resistance gene, or *aacC1*, the gentamicin resistance gene.

The prevalence of *nptII/aph(3’)-IIa* in publicly available *E. coli* and *Salmonella* genomes from pork and pig isolates collected as part of NARMS surveillance is described in Table 3. There were no records of *nptII* detection in *Enterococcus* or *Campylobacter* in the NARMS Global Resistome database. The prevalence of *nptII* in *E. coli* isolated from GalSafe™ pigs (1/55) in this study is similar to prevalence in conventional pigs over a 7-year period (17/1207) (Fisher’s exact test, *p* = 0.55).

**TABLE 3:** Prevalence of *nptII* (also known as *aph(3’)-IIa* in the NARMS database) as in publicly available *E. coli* and *Salmonella* genomes from pork and pig isolates collected as part of NARMS surveillance from 2013 to 2019.

NARMS resistome data	2013	2014	2015	2016	2017	2018	2019	Total
No. *aph(3’)-IIa* positive *E. coli*	0	2	1	0	5	3	6	17
Total No. *E. coli* genomes for year	0	63	2	1	238	399	504	1207
Percent *aph(3’)-IIa* positive *E. coli* (%)	–	3.2	50	0	2.1	0.75	1.2	1.41
No. *aph(3’)-IIa* positive *Salmonella*	1	2	1	3	4	7	19	37
Total no. *Salmonella* genomes for year	13	232	278	819	1333	1269	1498	5442
Percent *aph(3’)-IIa* positive *Salmonella* (%)	7.69	0.86	0.36	0.37	0.31	0.56	1.27	0.68

Whole genome sequence analysis of the *nptII*-positive *E. coli* showed that 3294 of 4.8 M usable reads aligned to the pPL657 reference sequence. Of those reads, ∼2700 contained *nptII* sequence and none of them had homology to the pPL657 used to create the GalSafe™ IGA or any porcine arm sequence (Supplementary Figure 1). BLAST analysis of the regions flanking the *nptII* gene revealed ∼5820 bp of homology to the entire *E. coli* Tn5 transposon (GenBank U00004.1), including Tn5: IS50, NeoR/KanR promoter, bleomycin resistance, and streptomycin resistance sequences. Alignment against the Tn5 transposon (GenBank U00004.1) provided further support, as ∼2700 reads aligned to the coordinates for *nptII* (1551–2345; Supplementary Figure 2). Together, this confirms that the *nptII* gene detected in the *E. coli* bacterial isolate resides on the Tn5 transposon mobile element found in natural bacterial reservoirs and is not a result of horizontal gene transfer from the GalSafe™ pig.

## Discussion

Our results show that antimicrobial resistance rates in GalSafe™ pigs are overall similar to those observed in conventional pigs. Antimicrobial resistance rates for some drugs, such as kanamycin in *Enterococcus*, were much lower in GalSafe™ pig isolates than in isolates collected by NARMS as part of national antimicrobial resistance surveillance. The prevalence of *nptII* in the target organisms isolated from GalSafe™ pigs was comparable to the prevalence in NARMS isolates.

In some instances, target organisms isolated from GalSafe™ pigs had higher rates of resistance than NARMS isolates, for example quinolones in *C. coli* (ciprofloxacin and nalidixic acid) and nitrofurantoin in *Enterococcus* spp. There are several potential explanations for this finding. It could be due to a unique environmental factor where the GalSafe™ pigs are housed or could be because gastrointestinal samples were collected from different parts of the intestinal tract in this antemortem study compared to the NARMS samples that are collected during processing (cecal specimens). Alternatively, there may be an emerging antibiotic resistance trend that has not yet been captured in the 2013–2019 NARMS data set. For example, recent NARMS data available on an online dashboard, but not yet in a downloadable dataset at the time of the study, indicates that *C. coli* resistance to ciprofloxacin and nalidixic acid increased from about 5% in 2013 to almost 30% by 2022 (Food and Drug Administration (FDA), 2025a). Another potential reason for the differences in resistance rates observed in *Enterococcus* may be due to the differences in species of *Enterococcus* spp. that contribute to the NARMS data versus the species recovered from the GalSafe™ pigs. For example, *E. faecalis* makes up 54% of the NARMS Enterococci but was not recovered from the GalSafe™ pigs, while *E. hirae* is less common in NARMS samples (20%) but had a high prevalence in the GalSafe™ pigs sampled in this study (75%).

Detection of *nptII* is not unusual in nature, complicating determination of the significance of detecting the gene in bacterial isolates from animals with the IGA. The *nptII* gene was present in 2.5% of bacterial clinical isolates resistant to kanamycin and neomycin collected between 1987 and 1991 in several European and Central and South American countries (European Food Safety Authority (EFSA), 2009). In addition, it has been estimated that 20%–40% (∼10^12^ bacteria) of the naturally occurring bacteria in animal or human digestive tracts are already resistant to kanamycin (Kaeppler, 2000; Nap et al., 1992; Ramessar et al., 2007). Genomic data from NARMS surveillance shows variable prevalence of *nptII* in porcine *E. coli* and *Salmonella* isolates over time, but the overall prevalence in *E. coli* (1.41%) isolated between 2013 and 2019 was similar to the *nptII* prevalence observed in this study, indicating that *nptII* prevalence has not increased in GalSafe™ pig *E. coli* compared to the level observed in standard pigs (Food and Drug Administration (FDA), 2025b). Genomic evaluation of the single *nptII*-positive *E. coli* isolated in this study was consistent with a natural origin of *nptII* as there was no sequence homology to the porcine arm sequences contained within the construct or the *nptII*-flanking sequences on plasmid that was used to create the IGA.

Resistance to kanamycin and streptomycin in *Enterococcus* spp. was lower in GalSafe™ pigs, potentially because no aminoglycosides are ever used in GalSafe™ pigs, reducing selection pressure on commensal fecal microbiota such as enterococci. Commercial swine operations represented in the NARMS data are not under any such restriction. Regular use of aminoglycoside antibiotics occurs in swine production and could contribute to the selection for resistant strains in some herds (Davies and Singer, 2020).

A limitation of this study is that a relatively small number of animals were sampled with a modest number of target organisms isolated from their fecal specimens. This is especially true for *Salmonella*, which was infrequently detected in GalSafe™ pigs and led to a limited comparison of resistance rates between GalSafe™ and conventional pigs. The low number of animals available for sampling also meant that several animals were sampled on more than one occasion. Therefore, ongoing surveillance is necessary to increase the sensitivity of detecting smaller differences in resistance rates between the two populations. Ongoing monitoring of *nptII* gene prevalence in bacterial isolates from GalSafe™ pigs can be used to assess the likelihood of horizontal gene transfer of the *nptII* gene from GalSafe™ genomic DNA to bacterial species associated with the GalSafe™ pig microbiota. Additionally, we relied on culture-based techniques and assessed only a limited number of isolates per fecal sample, which may have limited our ability to detect antimicrobial resistance present in low quantities in the sampled feces. Another limitation of the study is the reliance on NARMS data to create reference data to compare antimicrobial resistance rates observed in GalSafe™ pigs. Because we did not have access to wild type group of animals managed under the same conditions as GalSafe™ pigs, we relied on national surveillance data, which includes animals managed under a variety of husbandry conditions with various genetic backgrounds, leading to unobserved confounding of the data. Despite these differences, antimicrobial resistance rates in GalSafe™ pigs were often similar to what was observed in NARMS data.

In conclusion, we did not detect evidence of horizonal gene transfer of the *nptII* gene, which was the selectable genetic marker used in the creation of the GalSafe™ pig, to fecal organisms of potential human health importance (*E. coli*, *Salmonella*, *Campylobacter*, *Enterococcus)* under this study’s sampling conditions in this population of GalSafe™ pigs. Antimicrobial resistance rates and *E. coli nptII* prevalence in GalSafe™ pigs were similar to those observed in NARMS data. We did not detect any evidence that the presence of *nptII* in GalSafe™ pigs has contributed to antimicrobial resistance to date under current husbandry and antimicrobial use conditions.

## Data Availability

The raw data supporting the conclusions of this article will be made available by the authors, without undue reservation. NARMS analyzed in this study is available in public repositories (Food and Drug Administration (FDA), 2025a,b). Genomic data from this study can be found below: https://www.ncbi.nlm.nih.gov, BioProject PRJNA1466911.
