# Supplementary material for: Antimicrobial susceptibility patterns of commensal fecal bacteria isolated from pigs with an intentional genomic alteration that included the selectable marker gene nptII
Source: Front Microbiol. 2026 Aug 25;17:1885937. doi: 10.3389/fmicb.2026.1885937 (PMC13552116; doi:10.3389/fmicb.2026.1885937)

Supplemental Data

Supplementary Table 1. Primers used for multiplex PCR for antimicrobial resistance gene detection.

Primer ID	Primer sequence	Amplicon size	Target gene	Primer TM °C
**Bac16SF1**	5'-TCCTACGGGAGGCAGCAGT-3'	~850bp	Bacterial 16s	67
**Bac16SR1**	5'-CGTCATCCCCACCTTCCTCC-3'			70
**GenF**	5'-AGACGTAGCCACCTACTCCCAACAT-3'	~300bp	GmR/*aacC1*	68
**GenR**	5'-GGATCGTCACCGTAATCTGCTTGCA-3'			73
**AmpF**	5'-AAGTTCTGCTATGTGGCGCGGT-3'	~350bp	AmpR/*bla*	71
**AmpR**	5'-TGCGCAACGTTGTTGCCATTGCT-3'			77
**657F8762**	5'-CCCGGTTCTTTTTGTCAAGACC-3'	~350bp	neomycin/*nptII*	68
**657R9116**	5'-ATGCTCTTCGTCCAGATCATCC-3'			67
**KanF490**	5'-GGGTATAAATGGGCTCGCGA-3'	~650bp	KanR/*aph*	68
**KanR1143**	5'-GGAGAAAACTCACCGAGGCA-3'			67
**657F8580**	5'-TTGTATGGGATCTGATCTGGGGCCT -3'	~631bp	pPL657 IRES/NeoR	73

Supplementary Table 2. Comparison of antimicrobial resistance between GalSafe and NARMS isolates. Values represent resistant isolates (No. Resistant/Total N). Exact binomial tests were used to compare GalSafe resistance proportions to NARMS reference rates with 95% confidence intervals (CI). Fisher's exact tests were performed as sensitivity analyses; odds ratios presented are from Fisher's exact tests. P-values were adjusted within organism using the Holm method. A subset of this information, organism and drug combinations that were significantly different in the GalSafe population (adjusted binomial $p < 0.05$), is found in Table 2.

Organism	Drug	GalSafe	NARMS	GalSafe Resistance Proportion (95% CI)	OR	p (binomial)	Holm Adj p (binomial)	p (Fisher)	Holm Adj p (Fisher)
**Campylobacter coli**	**Ciprofloxacin**	**10/28**	**281/2131**	**0.357 (0.186–0.559)**	**3.65**	**0.00215**	**0.0172**	**0.00236**	**0.0189**
**Campylobacter coli**	**Nalidixic Acid**	**10/28**	**283/2131**	**0.357 (0.186–0.559)**	**3.62**	**0.00227**	**0.0172**	**0.00249**	**0.0189**
Campylobacter coli	Azithromycin	7/28	442/2131	0.250 (0.107–0.449)	1.27	0.64	1	0.638	1
Campylobacter coli	Erythromycin	7/28	531/2131	0.250 (0.107–0.449)	1.00	1	1	1	1
Campylobacter coli	Florfenicol	0/28	0/1820	0.000 (0.000–0.123)	0.00	1	1	1	1
Campylobacter coli	Tetracycline	23/28	1719/2131	0.821 (0.631–0.939)	1.10	1	1	1	1
Campylobacter coli	Gentamicin	0/28	20/2131	0.000 (0.000–0.123)	0.00	1	1	1	1
Campylobacter coli	Clindamycin	7/28	704/2131	0.250 (0.107–0.449)	0.68	0.427	1	0.424	1
**Enterococcus**	**Linezolid**	**1/48**	**0/3060**	**0.021 (0.001–0.111)**	**Inf**	**<0.001**	**<0.001**	**0.0154**	**0.154**
**Enterococcus**	**Tetracycline**	**7/48**	**2244/3060**	**0.146 (0.061–0.278)**	**0.06**	**<0.001**	**<0.001**	**<0.001**	**<0.001**
**Enterococcus**	**Nitrofurantoin**	**7/48**	**17/3060**	**0.146 (0.061–0.278)**	**30.37**	**<0.001**	**<0.001**	**<0.001**	**<0.001**
**Enterococcus**	**Erythromycin**	**2/48**	**1101/3060**	**0.042 (0.005–0.143)**	**0.08**	**<0.001**	**<0.001**	**<0.001**	**<0.001**
**Enterococcus**	**Kanamycin**	**0/48**	**359/2034**	**0.000 (0.000–0.074)**	**0.00**	**<0.001**	**0.00194**	**<0.001**	**0.0018**

Organism	Drug	GalSafe	NARMS	GalSafe Resistance Proportion (95% CI)	OR	p (binomial)	Holm Adj p (binomial)	p (Fisher)	Holm Adj p (Fisher)
**Enterococcus**	**Streptomycin**	**2/48**	**601/3060**	**0.042 (0.005–0.143)**	**0.18**	**0.00336**	**0.0336**	**0.00485**	**0.0534**
Enterococcus	Tigecycline	0/48	5/3060	0.000 (0.000–0.074)	0.00	1	1	1	1
Enterococcus	Daptomycin (<>E. faecium)	0/48	93/2736	0.000 (0.000–0.074)	0.00	0.413	1	0.408	1
Enterococcus	Daptomycin (=E. faecium)	0/48	15/324	0.000 (0.000–0.074)	0.00	0.173	1	0.234	1
Enterococcus	Gentamicin	0/48	163/3060	0.000 (0.000–0.074)	0.00	0.183	1	0.178	1
Enterococcus	Ciprofloxacin	3/48	139/3060	0.062 (0.013–0.172)	1.40	0.48	1	0.481	1
Enterococcus	Vancomycin	0/48	0/3060	0.000 (0.000–0.074)	0.00	1	1	1	1
Enterococcus	Ampicillin	0/48	0/1026	0.000 (0.000–0.074)	0.00	1	1	1	1
Enterococcus	Quinupristin/dalfopristin	26/48	2005/3060	0.542 (0.392–0.686)	0.62	0.128	1	0.125	1
Enterococcus	Chloramphenicol	3/48	304/3060	0.062 (0.013–0.172)	0.60	0.626	1	0.623	1
Escherichia	Kanamycin	9/55	21/238	0.164 (0.078–0.288)	2.02	0.0566	0.792	0.135	1
Escherichia	Trimethoprim/sulfamethoxazole	0/55	183/3237	0.000 (0.000–0.065)	0.00	0.0754	0.98	0.0727	1
Escherichia	Cefoxitin	0/55	115/3237	0.000 (0.000–0.065)	0.00	0.268	1	0.263	1

Organism	Drug	GalSafe	NARMS	GalSafe Resistance Proportion (95% CI)	OR	p (binomial)	Holm Adj p (binomial)	p (Fisher)	Holm Adj p (Fisher)
Escherichia	Azithromycin	0/55	25/3237	0.000 (0.000–0.065)	0.00	1	1	1	1
Escherichia	Chloramphenicol	1/55	195/3237	0.018 (0.000–0.097)	0.29	0.261	1	0.257	1
Escherichia	Tetracycline	31/55	2189/3237	0.564 (0.423–0.697)	0.62	0.0837	1	0.0826	1
Escherichia	Ceftriaxone	0/55	144/3237	0.000 (0.000–0.065)	0.00	0.179	1	0.174	1
Escherichia	Amoxicillin/clavulanic acid	0/55	119/3237	0.000 (0.000–0.065)	0.00	0.271	1	0.266	1
Escherichia	Ciprofloxacin	2/55	127/3237	0.036 (0.004–0.125)	0.92	1	1	1	1
Escherichia	Gentamicin	0/55	60/3237	0.000 (0.000–0.065)	0.00	0.629	1	0.626	1
Escherichia	Nalidixic Acid	0/55	59/3237	0.000 (0.000–0.065)	0.00	0.629	1	0.626	1
Escherichia	Meropenem	0/55	0/2295	0.000 (0.000–0.065)	0.00	1	1	1	1
Escherichia	Sulfisoxazole	0/55	0/3227	0.000 (0.000–0.065)	0.00	1	1	1	1
Escherichia	Ampicillin	9/55	683/3237	0.164 (0.078–0.288)	0.73	0.508	1	0.504	1
Salmonella	Cefoxitin	0/3	100/4243	0.000 (0.000–0.708)	0.00	1	1	1	1
Salmonella	Azithromycin	0/3	13/4243	0.000 (0.000–0.708)	0.00	1	1	1	1

Organism	Drug	GalSafe	NARMS	GalSafe Resistance Proportion (95% CI)	OR	p (binomial)	Holm Adj p (binomial)	p (Fisher)	Holm Adj p (Fisher)
Salmonella	Chloramphenicol	0/3	133/4233	0.000 (0.000–0.708)	0.00	1	1	1	1
Salmonella	Tetracycline	0/3	1303/4243	0.000 (0.000–0.708)	0.00	0.558	1	0.558	1
Salmonella	Ceftriaxone	0/3	112/4243	0.000 (0.000–0.708)	0.00	1	1	1	1
Salmonella	Amoxicillin/clavulanic acid	0/3	101/4243	0.000 (0.000–0.708)	0.00	1	1	1	1
Salmonella	Ciprofloxacin	0/3	158/4243	0.000 (0.000–0.708)	0.00	1	1	1	1
Salmonella	Gentamicin	0/3	71/4243	0.000 (0.000–0.708)	0.00	1	1	1	1
Salmonella	Nalidixic Acid	0/3	75/4243	0.000 (0.000–0.708)	0.00	1	1	1	1
Salmonella	Meropenem	0/3	0/2595	0.000 (0.000–0.708)	0.00	1	1	1	1
Salmonella	Sulfisoxazole	0/3	0/4243	0.000 (0.000–0.708)	0.00	1	1	1	1
Salmonella	Trimethoprim/sulfamethoxazole	0/3	54/4243	0.000 (0.000–0.708)	0.00	1	1	1	1
Salmonella	Ampicillin	0/3	400/4243	0.000 (0.000–0.708)	0.00	1	1	1	1
Salmonella	Kanamycin	0/3	13/551	0.000 (0.000–0.708)	0.00	1	1	1	1

Supplementary Table 3. Identification and quantification of species and genus for the bacterial isolates from fecal samples that were collected from GalSafe™ pigs on more than one occasion.

Animal ID	Date of collection	*E. coli*	*Salmonella*	*Enterococcus*		*Campylobacter*	
				faecium	*hirae*	*coli*	*lanienae*
1	4/4/2022	✓	-	-	✓	-	-
	8/8/2022	✓	-	✓	-	-	-
	9/26/2022	✓	-	-	✓	✓	-
2	4/4/2022	✓	-	-	✓	-	-
	9/26/2022	✓	-	-	✓	✓	-
3	4/4/2022	✓	-	✓	-	-	-
	9/26/2022	✓	-	-	✓	-	✓
4	6/6/2022	✓	-	-	✓	-	-
	8/8/2022	✓	-	✓	-	✓	-
5	6/6/2022	✓	-	-	✓	✓	-
	8/8/2022	✓	-	-	✓	✓	-
6	8/8/2022	✓	-	-	-	✓	-
	9/26/2022	✓	-	✓	-	-	-
7	8/8/2022	✓	-	-	-	-	-
	9/26/2022	✓	-	-	✓	-	-
	% of isolates recovered per genus	100%	0%	31%	69%	86%	14%

Key: ✓ indicates isolate recovered, - indicates isolate not recovered, % of isolates recovered per genus - # isolates per species divided by the total number of isolates for that genus.

Supplementary Figure 1. Integrative Genomics Viewer (IGV) image showing whole genome sequencing data from the one isolate positive for *nptII* aligned to the pPL657 sequence used for the IGA in GalSafe™ pigs. The *nptII* gene sequence in pPL657 is between positions 2239-3042. The 3' region of pPL657 (position 7591-7709) contains a repetitive sequence.

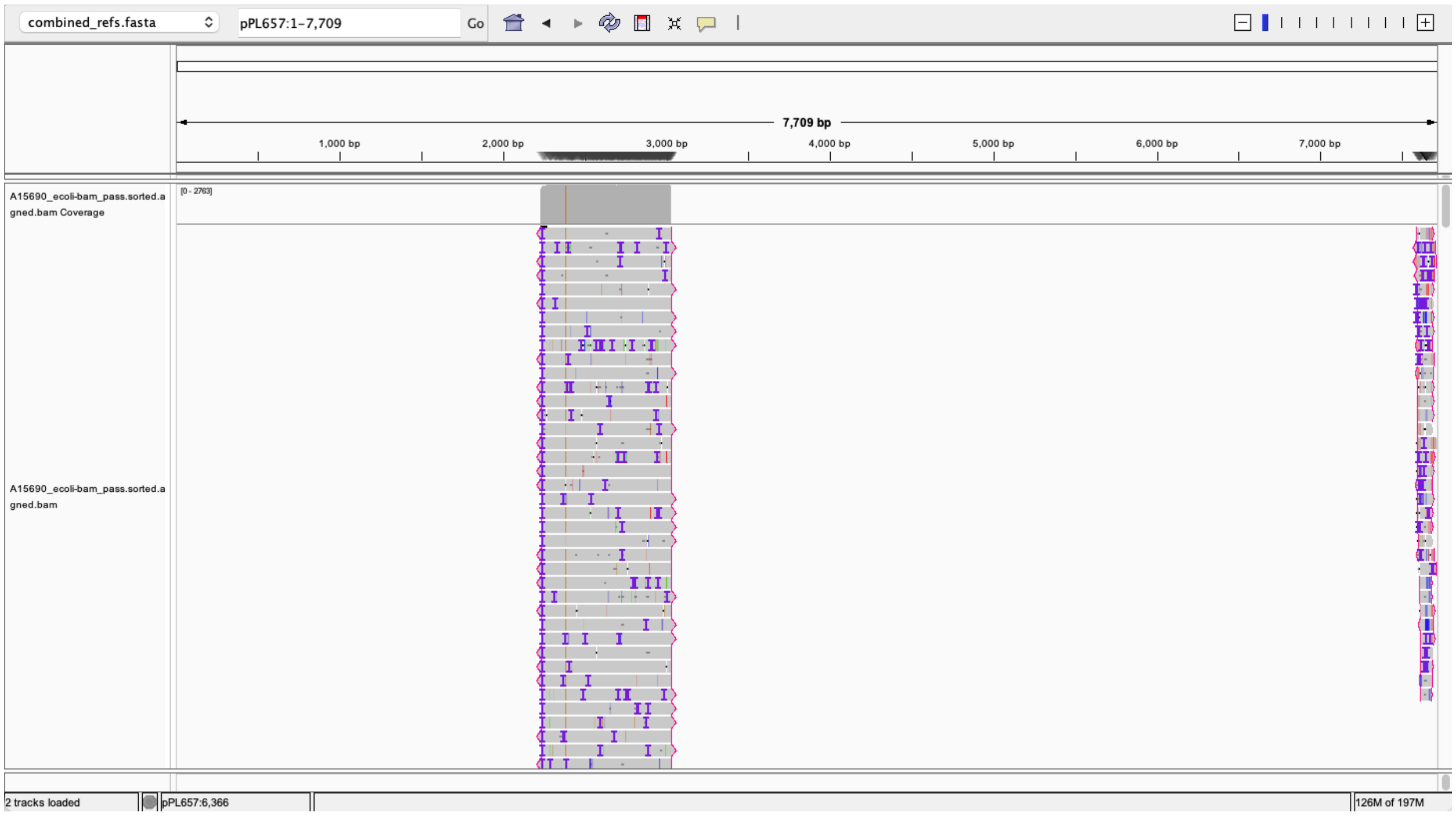

Supplementary Figure 2. Integrative Genomics Viewer (IGV) image showing whole genome sequencing data from the one isolate positive for *nptII* aligned to the GenBank U00004.1 sequence. The *nptII* gene sequence in GenBank U00004.1 is between positions 1551-2345. The Tn5 transposase is between positions 4297-5727.

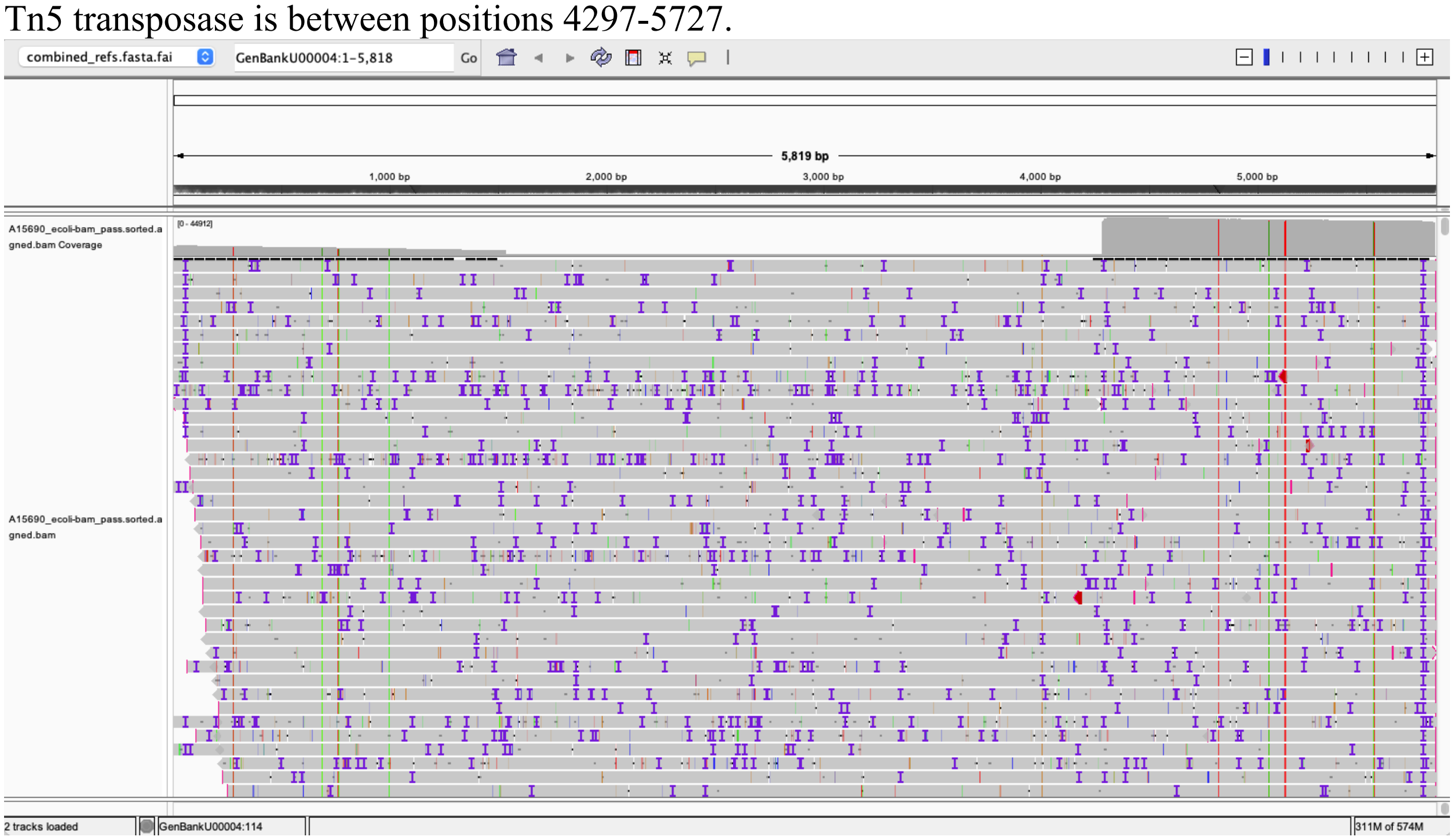

Supplement: Supplementary file 1 [file Data_Sheet_1.pdf]
